# Epac‐induced ryanodine receptor type 2 activation inhibits sodium currents in atrial and ventricular murine cardiomyocytes

**DOI:** 10.1111/1440-1681.12870

**Published:** 2017-12-07

**Authors:** Haseeb Valli, Shiraz Ahmad, Sujan Sriharan, Lydia D Dean, Andrew A Grace, Kamalan Jeevaratnam, Hugh R Matthews, Christopher L‐H Huang

**Affiliations:** ^1^ Physiological Laboratory University of Cambridge Cambridge UK; ^2^ Department of Biochemistry University of Cambridge Cambridge UK; ^3^ Faculty of Health and Medical Sciences University of Surrey Guildford Surrey UK; ^4^ PU‐RCSI School of Medicine Perdana University Serdang Selangor Darul Ehsan Malaysia

**Keywords:** 8‐(4‐chlorophenylthio)‐2′‐O‐methyladenosine‐3′,5′‐cyclic monophosphate, arrhythmic substrate, Ca^2+^ homeostasis, conduction velocity, dantrolene, Epac, Na^+^ current, ryanodine receptor

## Abstract

Acute RyR2 activation by exchange protein directly activated by cAMP (Epac) reversibly perturbs myocyte Ca^2+^ homeostasis, slows myocardial action potential conduction, and exerts pro‐arrhythmic effects. Loose patch‐clamp studies, preserving in vivo extracellular and intracellular conditions, investigated Na^+^ current in intact cardiomyocytes in murine atrial and ventricular preparations following Epac activation. Depolarising steps to varying test voltages activated typical voltage‐dependent Na^+^ currents. Plots of peak current against depolarisation from resting potential gave pretreatment maximum atrial and ventricular currents of −20.23 ± 1.48 (17) and −29.8 ± 2.4 (10) pA/μm^2^ (mean ± SEM [n]). Challenge by 8‐CPT (1 μmol/L) reduced these currents to −11.21 ± 0.91 (12) (*P* < .004) and −19.3 ± 1.6 (11) pA/μm^2^ (*P* < .04) respectively. Currents following further addition of the RyR2 inhibitor dantrolene (10 μmol/L) (−19.91 ± 2.84 (13) and −26.6 ± 1.7 (17)), and dantrolene whether alone (−19.53 ± 1.97 (8) and −27.6 ± 1.9 (14)) or combined with 8‐CPT (−19.93 ± 2.59 (12) and −29.9 ± 2.5(11)), were indistinguishable from pretreatment values (all *P *>> .05). Assessment of the inactivation that followed by applying subsequent steps to a fixed voltage 100 mV positive to resting potential gave concordant results. Half‐maximal inactivation voltages and steepness factors, and time constants for Na^+^ current recovery from inactivation in double‐pulse experiments, were similar through all the pharmacological conditions. Intracellular sharp microelectrode membrane potential recordings in intact Langendorff‐perfused preparations demonstrated concordant variations in maximum rates of atrial and ventricular action potential upstroke, (d*V*/d*t*)_max_. We thus demonstrate an acute, reversible, Na^+^ channel inhibition offering a possible mechanism for previously reported pro‐arrhythmic slowing of AP propagation following modifications of Ca^2+^ homeostasis, complementing earlier findings from chronic alterations in Ca^2+^ homeostasis in genetically‐modified *RyR2‐*P2328S hearts.

## 1 INTRODUCTION

Cardiac arrhythmias result from disruptions in the normal excitable activity propagating through successive structures in the heart. They arise from altered function in surface membrane ionic channels whose successive activation and inactivation underlies the production and propagation of cardiac action potentials (AP). Sustained arrhythmia may require both triggering events and arrhythmic substrate to maintain the resulting abnormal electrical activity.^1^, ^2^, ^3^ Arrhythmic substrate arises either from slowed myocardial AP conduction or activation, exemplified by Brugada Syndrome,^4^, ^5^ or altered AP recovery, reflected in altered AP duration and/or refractoriness, exemplified by long QT syndrome.^6^ Arrhythmic risk is also associated with dysregulated intracellular Ca^2+^ homeostasis. This may arise from abnormal ryanodine receptor‐2 (RyR2)‐mediated sarcoplasmic reticular Ca^2+^ release. The resulting elevations in diastolic [Ca^2+^] and spontaneous propagated intracellular Ca^2+^ waves increase electrogenic Na^+^‐Ca^2+^ exchanger activity thereby driving delayed after‐depolarisations that may trigger premature ventricular beats.^7^

Murine hearts carrying genetically altered RyR2‐Ca^2+^ release channel and SR Ca^2+^ storage protein calsequestrin‐2 have successfully modelled such triggering events. They recapitulate mutations associated with the chronic pro‐arrhythmic condition of human catecholaminergic polymorphic ventricular tachycardia (CPVT).^8^, ^9^ Triggering events have also been reported following acute adrenergic activation produced by modifications of RyR2‐mediated SR Ca^2+^ release^10^ and surface Ca^2+^ channel properties in *wild‐type* hearts expressing *normal* RyR2 and calsequestrin‐2.^11^, ^12^ More recent studies selectively activated RyR2‐Ca^2+^ release channels using the phosphokinase A (PKA)‐independent exchange protein directly activated by cAMP (Epac) pathway.^13^, ^14^, ^15^ This increased Ca^2+^ spark frequencies in adult rat cardiac myocytes^16^ and amplitudes of Ca^2+^‐dependent Ca^2+^ release after isoproterenol treatment^17^ in murine ventricular cardiomyocytes. They also increased the amplitudes and frequencies of spontaneous Ca^2+^ release.^18^ These changes correlated with increases in triggered activity and ventricular tachycardia (VT) in murine hearts.^18^

Fewer studies have explored arrhythmic substrate under conditions of altered Ca^2+^ homeostasis. Neither chronic modifications in Ca^2+^ homeostasis in *RyR2‐P2328S* models nor acute manipulations of Ca^2+^ homeostasis in WT hearts altered AP recovery characteristics as reflected in AP durations (APD), refractory periods (ERP), or the relationships between these.^8^, ^12^, ^19^ However, murine *RyR2‐P2328S* CPVT cardiac models showed reduced atrial^20^ and ventricular conduction velocities in common with Na_v_1.5‐haploinsufficient *Scn5a+/−* hearts modelling the Brugada Syndrome.^21^ Pharmacological inhibition of RyR2‐mediated Ca^2+^ release with flecainide partly rescued these effects.^22^, ^23^, ^24^, ^25^ Furthermore, selective, acute RyR2 activation through the Epac pathway produced parallel pro‐arrhythmic effects.^18^ It correspondingly produced decreases in AP conduction velocities that were partially reversed by the RyR2 antagonist dantrolene, with an absence of alterations in AP recovery characteristics.^19^

The mechanism for the conduction velocity changes in *RyR2‐P2328S* hearts was identified as the direct action of intracellular Ca^2+^ on Na_v_1.5 function^20^, ^21^, ^26^ and/or Na_v_1.5 membrane expression.^27^ However, the mechanisms by which ***acute*** manipulations of intracellular Ca^2+^ homeostasis, particularly Epac activation, alter AP conduction have not been investigated. The present experiments assessed Nav1.5 activation, inactivation, and recovery from inactivation following acute rather than chronic manipulations of Ca^2+^ homeostasis, and in WT rather than genetically‐modified hearts. They employed the loose patch technique for voltage‐clamping of Na^+^ current. This apposes an electrode containing extracellular solution against an intact cell surface membrane without accessing intracellular space. Studies were thus performed in cardiomyocytes in intact murine atrial and ventricular preparations without perturbing extracellular [Na^+^] and intracellular Ca^2+^ homeostasis^21^, ^28^, ^29^ as opposed to following cardiomyocyte isolation necessitated by conventional whole‐cell patch clamp techniques.^30^, ^31^

Recent cardiomyocyte studies involving reversible manipulations of loose patch pipette [Na^+^] had identified early inward currents in response to step depolarisations with Na^+^ currents responsible for AP conduction and the maximum upstroke rate, (d*V*/d*t*)_max_, of the cardiac action potential.^21^ The corresponding changes in such (d*V*/d*t*)_max_ were accordingly determined by independent experiments performing intracellular sharp electrode recordings of membrane potential in intact atrial and ventricular preparations. These explored the extent to which changes in observed (d*V*/d*t*)_max_, paralleled corresponding changes in Na^+^ currents in the loose‐patch experiments with similar pharmacological manipulations. Such correlations would be consistent with previously reported relationships between (d*V*/d*t*)_max_ and peak Na^+^ currents (*I*_Na_).^32^

We thus studied electrophysiological effects of pharmacological manipulations of Ca^2+^ homeostasis through Epac activation on Na^+^ currents in a near physiological environment for the first time. The Epac activator (8‐pCPT‐2′‐O‐Me‐cAMP: 8‐(4‐chlorophenylthio)‐2′‐O‐methyladenosine‐3′,5′‐cyclic monophosphate)^13^ at approximately 1 μmol/L offered 300‐fold preferential selectivity for Epac over PKA action.^13^, ^33^, ^34^, ^35^ The RyR2 antagonist dantrolene inhibits RyR2‐mediated diastolic Ca^2+^ release, decreases frequencies and durations of aberrant Ca^2+^ sparks in myocyte cultures modelling CPVT^36^ and cardiac failure^37^, ^38^ and increases thresholds for Ca^2+^‐induced sarcoplasmic reticular Ca^2+^‐release in myocytes from failing but not normal rabbit hearts.^38^ Thus, both 8‐CPT and dantrolene act on intracellular targets following access across cardiomyocyte surface membrane as a whole whether during loose‐patch or sharp electrode recordings. Finally, the results obtained with the approach adopted here could be directly compared to corresponding explorations in intact Langendorff perfused murine hearts. In addition to establishing the expected alterations in cellular Ca^2+^ homeostasis, they used identical Epac activators and RyR2 antagonists in identical pharmacological protocols exploring the effects of Epac activation and their reversibility applying these to examine changes in AP conduction velocity and arrhythmic substrate.^18^, ^19^

## 2 RESULTS

### 2.1 Currents obtained in the combined pulse procedure

**Figure 1:**
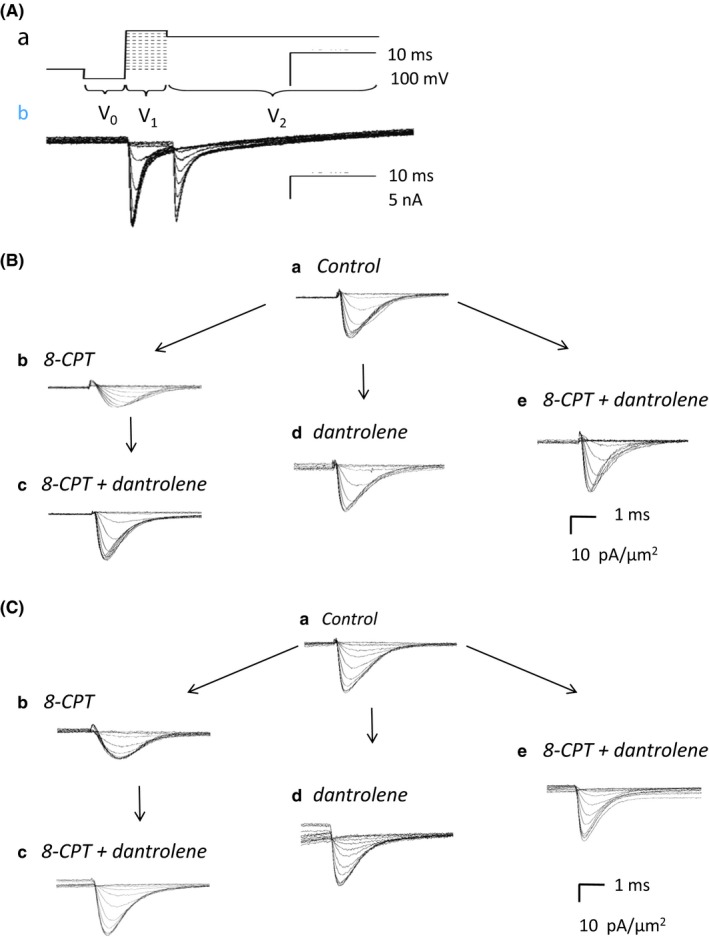
Ionic currents from an isolated atrial preparation studied under loose‐patch clamp. (Aa) Pulse protocol designed to investigate Na^+^ channel activation and inactivation. (Ab) Typical recordings obtained using with a pipette of 28 μm tip diameter. Inward currents in nA are represented as negative. All currents shown in subsequent panels converted to current density in units of (pA/μm^2^) to account for pipette diameters, typically 28‐32 μm. (B, C) Na^+^ currents observed upon (B) activation by depolarisation to a level *V*_1_, before and following pharmacological challenge and (C) in response to the voltage step from levels *V*_1_ to the final level *V*_2_. The latter permitted assessment of Na^+^ channel inactivation produced by the voltage step to level *V*_1_. Recordings made (a) under pretreatment conditions, when no drug was present, (b) in the presence of 8‐CPT (1 μmol/L), (c) in the presence of a combination of 8‐CPT (1 μmol/L) and dantrolene (10 μmol/L) after the addition of 8‐CPT, (d) after the addition of dantrolene (10 μmol/L), and (e) in the presence of a combination of 8‐CPT (1 μmol/L) and dantrolene (10 μmol/L). (d) and (e) were obtained following direct introduction of pharmacological agents following pretreatment conditions in the absence of any preceding application of 8‐CPT

**Figure 2:**
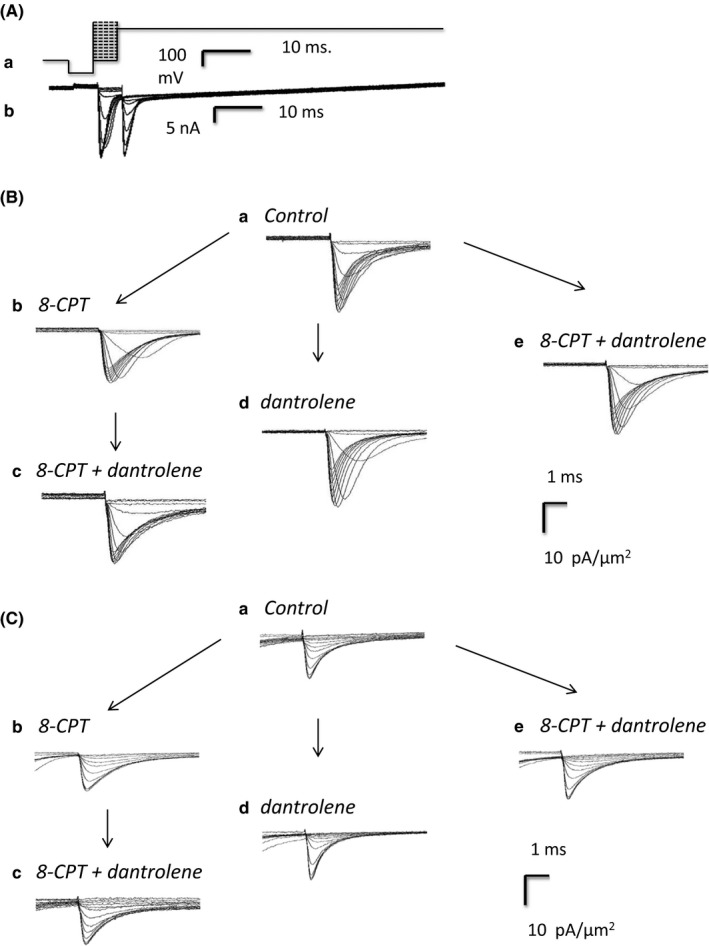
Ionic currents from an isolated ventricular preparation studied under loose‐patch clamp. (Aa) Pulse protocol designed to investigate Na^+^ channel activation and inactivation. (Ab) Typical recordings obtained using with a pipette of tip diameter 30 μm. Inward currents in nA represented as negative. All currents shown in subsequent figures have been converted to current density in units of (pA/μm^2^) to account for pipette diameters, typically 28‐32 μm. (B, C) Na^+^ currents observed upon (B) activation by depolarisation to a level *V*_1_, before and following pharmacological challenge and (C) in response to the voltage step from levels *V*_1_ to the final level *V*_2_. The latter permitted assessment of Na^+^ channel inactivation produced by the voltage step to level *V*_1_. Recordings made (a) under pretreatment conditions, when no drug was present, (b) in the presence of 8‐CPT (1 μmol/L), (c) in the presence of a combination of 8‐CPT (1 μmol/L) and dantrolene (10 μmol/L) after the addition of 8‐CPT, (d) after the addition of dantrolene (10 μmol/L), and (e) in the presence of a combination of 8‐CPT (1 μmol/L) and dantrolene (10 μmol/L). (d) and (e) were obtained following direct introduction of pharmacological agents following pretreatment conditions in the absence of any preceding application of 8‐CPT

Figures 1 and 2 illustrate results obtained from the isolated atrial (Figure 1) and ventricular (Figure 2) preparations subject to a combined pulse procedure (Panel Aa) designed to explore both activation and inactivation properties of Na^+^ currents. Cells were first held at the resting membrane potential (RMP) for 5 ms from the beginning of the recording period. A prepulse of duration 5 ms to *V*_0_ = (RMP−40) mV was then applied to remove any residual Na^+^ current inactivation and standardise the initial activation state of Na^+^ channels within the patch. Na^+^ current activation properties were then investigated using the initial depolarising test voltage steps made to a voltage varied with the 13 successive sweeps between *V*_1_ = RMP to (RMP + 120) mV in +10 mV increments. These currents, following correction for residual leakage by a P/4 protocol, provided a family of records reflecting the voltage dependence of Na^+^ channel activation (Panels Ab and Ba). Inward currents are represented as downward, negative deflections. Records typically began with a consistent small upward deflection in response to the −40 mV prepulse. The subsequent voltage steps to level *V*_1_ yielded a family of inward currents initially increasing with time to a peak value that increased as *V*_1_ was made more positive. This was followed by a decay reflecting channel inactivation whose extent and kinetics was similarly determined by the voltage *V*_1_.

The subsequent voltage steps in the pulse protocol were used to assess properties of the Na^+^ current inactivation resulting from the voltage step to *V*_1_. Thus, 5 ms following imposition of the latter step, an additional step was applied to a fixed voltage of *V*_2_ = (RMP + 100) mV that would result in a peak Na^+^ current reflecting the extent of the preceding channel inactivation (Panels Ab and Ca). The step to voltage *V*_2_ thus elicited a second family of currents which decreased in amplitude with increasing *V*_1_. Both sets of responses showed nonlinear increments in the magnitude of currents obtained at successive voltages reflecting the nonlinear dependence of either activation or inactivation about the voltage *V*_1_.

### 2.2 Currents reflecting Na^+^ channel activation

Figures 1B and 2B show typical families of current records illustrating Na^+^ current activation obtained in response to the initial steps between voltages *V*_0_ and *V*_1_ in atrial (Figure 1) and ventricular (Figure 2) preparations. In both panels B and C currents are normalised to the area subtended by the pipette tip lumen (in μm^2^) to give current densities. Recordings were made before (pretreatment: Panel Ba), and following challenge by 8‐CPT (1 μmol/L) (Bb), and then 8‐CPT (1 μmol/L) combined with dantrolene (10 μmol/L) (Bc). These were compared with records obtained following challenge by dantrolene (10 μmol/L) alone (Bd) and in combination with 8‐CPT (1 μmol/L) (Be).

The pretreatment Na^+^ currents showed typical activation and inactivation time courses with their amplitude nonlinearly graded with the size of the depolarising step (Ba). 8‐CPT markedly decreased the Na^+^ current amplitudes at all test voltages in both atrial and ventricular preparations (Bb). However, further inclusion of dantrolene in the test solutions rescued the Na^+^ current, reversing the inhibitory action of 8‐CPT alone (Bc). These findings were corroborated by another control procedure. Thus, families of currents before treatment (Ba) were recorded and the pretreatment solution then replaced by one including dantrolene either alone (Bd) or combined with 8‐CPT (Be). All these conditions (Ba, Bd, Be) gave similar Na^+^ currents. Thus, 8‐CPT reduces Na^+^ current in both atrial and ventricular preparations, but this action was reversed or absent in the presence of dantrolene whether by itself or in combination with 8‐CPT.

### 2.3 Currents reflecting Na^+^ channel inactivation

Records demonstrating typical families of Na^+^ current inactivation from atrial and ventricular preparations are shown in Figures 1C and 2C. Each family of records was obtained from an individual patch in response to the steps between the voltages *V*_1_ and *V*_2_ (panel Aa). These illustrate the extent of Na^+^ channel inactivation resulting from the step to voltage *V*_1_. Only the fraction of channels spared such inactivation would then be activated by the step to the fixed voltage *V*_2_. The Na^+^ current amplitudes would therefore permit quantification of the voltage dependence of Na^+^ channel inactivation at the voltage *V*_1_. Records were obtained before 8‐CPT challenge (pretreatment) (Ca), following 8‐CPT challenge (Cb), and following challenge by 8‐CPT combined with dantrolene (Cc). They were compared with results of transferring pretreatment preparations directly to solutions containing dantrolene alone (Cd) or dantrolene combined with 8‐CPT (Ce). The resulting Na^+^ currents showed typical activation and inactivation time courses, but these decreased in amplitude the more depolarised the voltage *V*_1_ (Ca). Again, 8‐CPT markedly decreased Na^+^ current amplitudes at all voltages tested (Cb), and further inclusion of dantrolene restored the Na^+^ currents (Cc). Na^+^ currents following challenge by dantrolene alone (Cd) or dantrolene in combination with 8‐CPT (Ce) were similar in amplitude and waveform as pretreatment records. Thus, the pulse procedures assessing current inactivation similarly demonstrated that 8‐CPT reduced Na^+^ current amplitude and this action was rescued by further addition of dantrolene.

### 2.4 Voltage dependences of atrial Na^+^ current activation and inactivation

**Figure 3:**
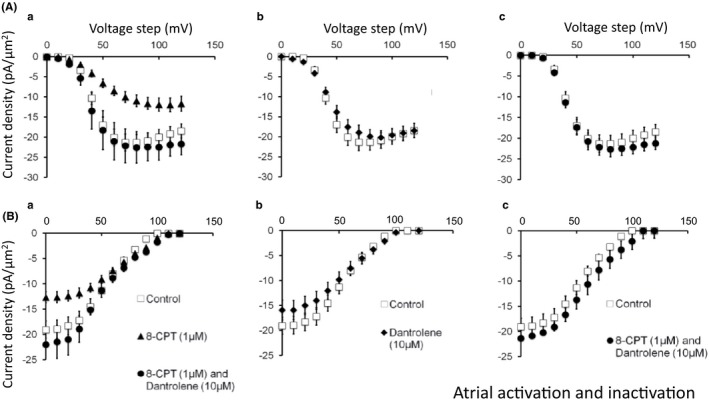
Voltage dependence of atrial Na^+^ current activation and inactivation under the different pharmacological conditions employing 8‐CPT and dantrolene. Dependences of (A) Na^+^ current activation (mean ± SEM) upon voltage step *V*_1_, before (pretreatment) and following pharmacological challenge and (B) Na^+^ current inactivation (mean ± SEM) as a function of voltage step *V*_1_, assessed using the further voltage step *V*_2_, before and after pharmacological challenge. Results obtained (a) before (open squares) and following introduction of 8‐CPT (filled triangles) and a combination of 8‐CPT and dantrolene (filled circles), (b) before (open squares) and following introduction of dantrolene (filled diamonds), (c) before (open squares) and following introduction of a combination of 8‐CPT and dantrolene (filled circles)

**Figure 4:**
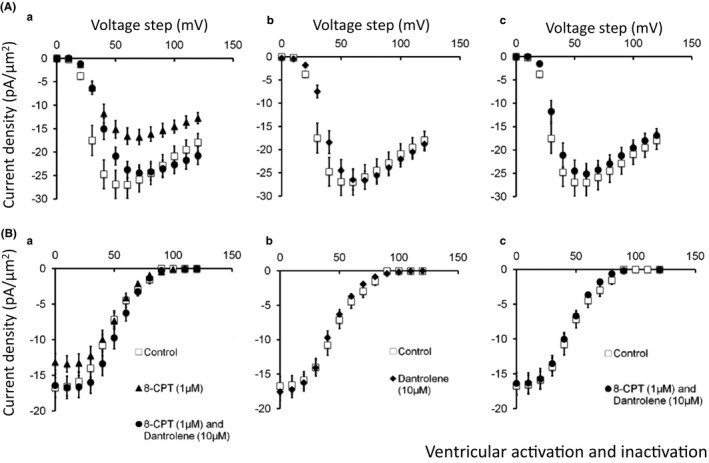
Voltage dependence of ventricular Na^+^ current activation and inactivation under the different pharmacological conditions employing 8‐CPT and dantrolene. Dependences of (A) Na^+^ current activation (mean ± SEM) upon voltage step V_1_, before (pretreatment) and following pharmacological challenge and (B) Na^+^ current inactivation (mean ± SEM) as a function of voltage step *V*_1_ assessed using the further voltage step *V*_2_, before and after pharmacological challenge. Results obtained (a) before (open squares) and following introduction of 8‐CPT (filled triangles) and a combination of 8‐CPT and dantrolene (filled circles), (b) before (open squares) and following introduction of dantrolene (filled diamonds), (c) before (open squares) and following introduction of a combination of 8‐CPT and dantrolene (filled circles)

Figures 3 and 4 illustrate voltage‐dependences of atrial (Figure 3) and ventricular (Figure 4) Na^+^ current activation (A) and inactivation (B) under each of the experimental conditions described above (a–c). They plot the peak Na^+^ current (mean ± standard error of the mean [SEM]) against the voltage *V*_1_. Results are shown for pretreatment conditions (open symbols) and following addition of 8‐CPT (filled triangles) and 8‐CPT combined with dantrolene (filled circles) (a). These results were compared with findings obtained before and following addition of dantrolene alone (pretreatment: open symbols; dantrolene: filled diamonds) (b) and before and following addition of dantrolene in combination with 8‐CPT (pretreatment: open symbols; 8 CPT with dantrolene, filled circles) (c). In both atrial (Figure 3) and the ventricular preparations (Figure 4) peak current increased with the amplitudes of depolarising steps exceeding +10 mV in size to a maximum value at a voltage excursion around +80 mV. They then decreased with further depolarisation as expected with approach of V_1_ towards the Na^+^ current reversal potential. Table 1 summarizes the results of applying voltage steps of a fixed +80 mV amplitude, employing 12 signal‐averaged records, to explore the effect of the different pharmacological agents on such maximum peak current under the different pharmacological conditions. The presence of significant differences in both atrial (*F* = 3.462, *P* = .013) and ventricular findings between experimental groups (*F* = 4.071, *P* = .006) prompted pairwise testing demonstrating differences between results of exposure to 8‐CPT compared to findings from untreated patches in both atrial (*P* < .04) and ventricular cardiomyocytes (*P* < .004; α = .05). In contrast, there were no detectable differences (*P *>* *.2) of findings obtained with challenge by 8‐CPT followed by 8‐CPT combined with dantrolene, and dantrolene whether alone, or combined with 8‐CPT, when all were compared from data from untreated patches.

**Table 1:** Maximum inward currents in atrial and ventricular preparations under the tested pharmacological conditions

Pharmacological condition	Maximum currents, (Mean ± SEM (n–value), pA/μm^2^	
	Atrial preparation	Ventricular preparation
(a) Untreated	−20.23 ± 1.48 (n = 17)***	−29.8 ± 2.4 (n = 10)**
(b) 8–CPT	−11.21 ± 0.91 (n = 12)***	−19.3 ± 1.6 (n = 11)**
(c) 8–CPT followed by 8–CPT + dantrolene	−19.91 ± 2.84 (n = 13)	−26.6 ± 1.7 (n = 17)
(d) Dantrolene alone	−19.53 ± 1.97 (n = 8)	−27.6 ± 1.9 (n = 14)
(e) 8–CPT + dantrolene	−19.93 ± 2.59 (n = 12)	−29.9 ± 2.5 (n = 11)

8–(4–chlorophenylthio) adenosine 3′,5′–cyclic monophosphate (8–CPT, 1 μmol/L); dantrolene (10 μmol/L). One‐way ANOVA: atrial preparations: *F* = 3.462, *P* = .013; ventricular preparations: *F* = 4.071, *P *=* *.006. Posthoc comparisons (after Bonferroni correction for four pairwise tests): *^*^ *P* < .04; *^**^ *P* < .004, relative to untreated group; no significant changes in all remaining groups (c)–(e) compared to control: *P* > .2.

These findings from Table 1 were corroborated by the corresponding atrial and ventricular Na^+^ current‐voltage curves. Peak atrial currents increased with depolarisation to approximately −18.5 ± 1.8 pA/μm^2^ (n = 13) (Figure 3Aa) and −13.5 ± 1.3 pA/μm^2^ (n = 14) before and following 8‐CPT challenge. In contrast, currents following further addition of dantrolene (−22.5 ± 3.9 pA/μm^2^ [n = 6]) (Aa), or exposure to dantrolene either alone (−20.2 ± 1.9 pA/μm^2^ [n = 11]) (Ab), or combined with 8‐CPT (−22.7 ± 1.9 pA/μm^2^ [n = 7]) (Ac) were similar to pretreatment values. Similar results emerged from atrial Na^+^ current inactivation studies of peak Na^+^ current in response to voltage steps from *V*_1_ to the fixed level *V*_2,_ plotted against inactivating voltage *V*_1_ (Figure 3B). Maximum currents at the most negative *V*_1_ before treatment (−19.1 ± 1.7 pA/μm^2^ [n = 13]) fell (to −13.6 ± 0.9 pA/μm^2^ [n = 14] (*P* < .01)) with 8‐CPT challenge, whereas currents observed following further addition of dantrolene (−22.0 ± 3.3 pA/μm^2^ [n = 5]) (Ba), or exposure to dantrolene whether alone (Bb), or combined with 8‐CPT (Bc) (−19.4 ± 1.8 pA/μm^2^ [n = 11] and −21.4 ± 1.8 pA/μm^2^ [n = 8] respectively) approximated pretreatment values. These changes occurred in an absence of alterations in fits of a Boltzmann function to the inactivation curves. Neither half‐maximal voltages, V* (54.9 ± 2.1 mV [pretreatment], 55.8 ± 2.1 mV (8‐CPT); 55.7 ± 6.8 mV [addition of dantrolene to 8‐CPT]; 55.7 ± 3.2 mV [dantrolene] and 58.6 ± 2.2 mV [dantrolene with 8‐CPT]), nor steepness factors, *k*, (13.2 ± 0.6 mV, 15.3 ± 0.7 mV, 14.4 ± 0.83 mV, 14.1 ± 0.6 mV, and 15.1 ± 0.5 mV respectively) showed significant differences between groups.

### 2.5 Voltage dependences of ventricular Na^+^ current activation and inactivation

Current‐voltage curves describing voltage dependences of ventricular Na^+^ current activation (Figure 4A) similarly corroborate results in Table 1. Peak Na^+^ currents increased with depolarisation to −27.0 ± 2.8 pA/μm^2^ (n = 11) (Figure 4Aa) under pretreatment conditions but only to −16.8 ± 1.6 pA/μm^2^ (n = 10) in the presence of 8‐CPT. However, currents following further addition of dantrolene (−24.4 ± 1.7 pA/μm^2^ [n = 10]) (Aa), or exposure to dantrolene whether alone (−26.6 ± 2.0 pA/μm^2^) (n = 12) (Ab), or combined with 8‐CPT (−25.1 ± 2.2 pA/μm^2^) (n = 11) (Ac) approximated pretreatment values. Protocols exploring Na^+^ current inactivation with voltage *V*_1_, reflected in currents observed at voltage *V*_2_ demonstrated currents at the most negative *V*_1_ before treatment (−16.7 ± 1.4 pA/μm^2^ [n = 7]) falling (to −13.1 ± 1.1 pA/μm^2^ [n = 9]) with 8‐CPT challenge. However, currents following further addition of dantrolene (−16.4 ± 1.7 pA/μm^2^ [n = 7]) (Ba), or exposure to dantrolene whether alone (Bb), or combined with 8‐CPT (Bc) (−17.6 ± 1.2 pA/μm^2^ [n = 12] and −16.3 ± 1.6 pA/μm^2^ [n = 8] respectively) approximated pretreatment values (all *P* > .05). These changes occurred in an absence of alterations in fits of a Boltzmann function to the inactivation curves. Neither V* nor k showed significant differences between experimental groups (46.0 ± 3.5, 11.2 ± 1.4 [pretreatment]; 50.3 ± 2.3, 10.0 ± 0.4 [8‐CPT]; 53.4 ± 2.1, 9.8 ± 0.5 [addition of dantrolene to 8‐CPT]; 42.5 ± 1.4, 11.7 ± 0.6 [dantrolene alone] and 45.6 ± 1.9, 10.8 ± 0.5 mV [dantrolene with 8‐CPT]).

### 2.6 Time course of Na^+^ channel recovery from inactivation

**Figure 5:**
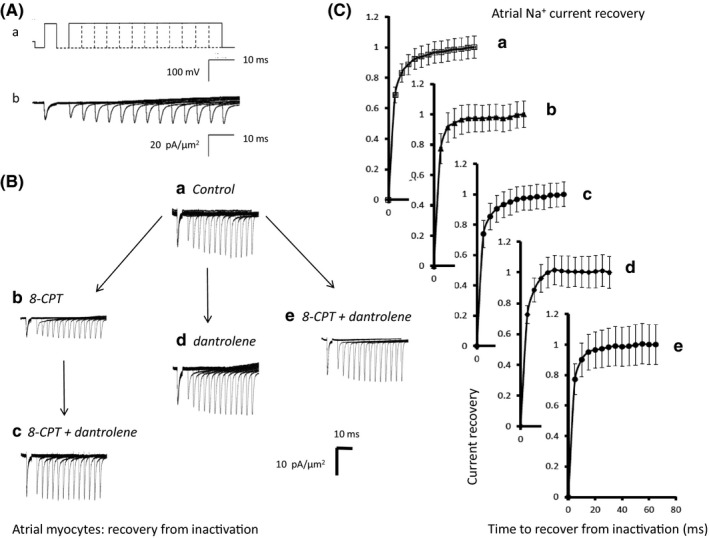
Time course of atrial Na^+^ current recovery from inactivation. (Aa) Pulse protocols used to investigate the time course of Na^+^ current recovery from inactivation (Ab) Recovery of Na^+^ currents with time after inactivation. A pipette of tip diameter 28 μm was used to obtain the currents shown in (b). (B) Families of recovery currents recorded (a) when no drug was present (pretreatment), (b) in the presence of 8‐CPT (1 μmol/L), (c) in the presence of a combination of 8‐CPT (1 μmol/L) and dantrolene (10 μmol/L) after the addition of 8‐CPT, (d) after the addition of dantrolene (10 μmol/L), and (e) in the presence of a combination of 8‐CPT (1 μmol/L) and dantrolene (10 μmol/L). (d) and (e) were obtained following direct introduction of pharmacological agents following pretreatment conditions in the absence of any preceding application of 8‐CPT. (C) Dependences of Na^+^ current recovery (mean ± SEM) with recovery time before and following pharmacological challenge. The graphs show results (a) before and (b) following introduction of 8‐CPT followed by (c) a combination of 8‐CPT and dantrolene, (d) dantrolene alone and (e) a combination of 8‐CPT and dantrolene

**Figure 6:**
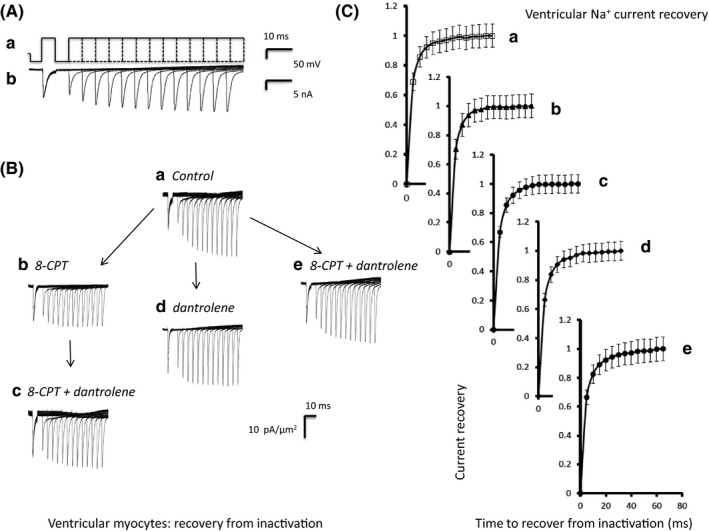
Time course of ventricular Na^+^ current recovery from inactivation. (Aa) Pulse protocols used to investigate the time course of Na^+^ current recovery from inactivation (Ab) Recovery of Na^+^ currents with time after inactivation. A pipette of tip diameter 28 μm was used to obtain the currents shown in (b). (B) Families of recovery currents recorded (a) when no drug was present (pretreatment), (b) in the presence of 8‐CPT (1 μmol/L), (c) in the presence of a combination of 8‐CPT (1 μmol/L) and dantrolene (10 μmol/L) after the addition of 8‐CPT, (d) after the addition of dantrolene (10 μmol/L), and (e) in the presence of a combination of 8‐CPT (1 μmol/L) and dantrolene (10 μmol/L). (d) and (e) were obtained following direct introduction of pharmacological agents following pretreatment conditions in the absence of any preceding application of 8‐CPT. (C) Dependences of Na^+^ current recovery (mean ± SEM) with recovery time before and following pharmacological challenge. The graphs show results (a) before and (b) following introduction of 8‐CPT followed by (c) a combination of 8‐CPT and dantrolene, (d) dantrolene alone and (e) a combination of 8‐CPT and dantrolene

The subsequent protocols investigated timecourse of Na^+^ channel recovery from inactivation following atrial or ventricular repolarisation (Figures 5A and 6A) under the same set of pharmacological conditions as described above. The voltage was held at the RMP for 1 ms from the beginning of the recording period before imposition of a hyperpolarising prepulse to voltage *V*_0_ = (RMP−40) mV for 4 ms. This established a consistent baseline level of Na^+^ current inactivation as in the previous protocol (Figures 5 and 6; panel Aa). A P1 conditioning step between *V*_0_ and *V*_1_ = (RMP + 80) mV of 5 ms duration then elicited activation of an initial Na^+^ current followed by its inactivation decay (Figures 5 and 6, panel Ab, B). Depolarising P2 steps of 5 ms duration to voltage *V*_3_ = (RMP + 80) mV were then imposed following different time intervals, T, which varied between 5 to 65 ms in 5 ms increments through the 12 successive sweeps making up the protocol. These P2 steps could be used to assess the recovery with time of the peak Na^+^ current from inactivation (Ab). The peak currents are normalized to their values obtained in the P1 step (Figures 5C and 6C). Fits of time constants to this recovery gave statistically indistinguishable results (*P *>* *.05) in each case (a)–(e) (atria: 4.58 ± 0.31 ms; 3.99 ± 0.38 ms; 3.90 ± 0.22 ms; 3.92 ± 0.38 ms and 3.91 ± 0.25 ms; ventricles: 4.6 ± 0.4 ms; 4.5 ± 0.3 ms; 4.7 ± 0.2 ms; 4.7 ± 0.3 ms and 4.8 ± 0.3 ms, respectively).

### 2.7 Microelectrode recordings of AP characteristics

**Figure 7:**
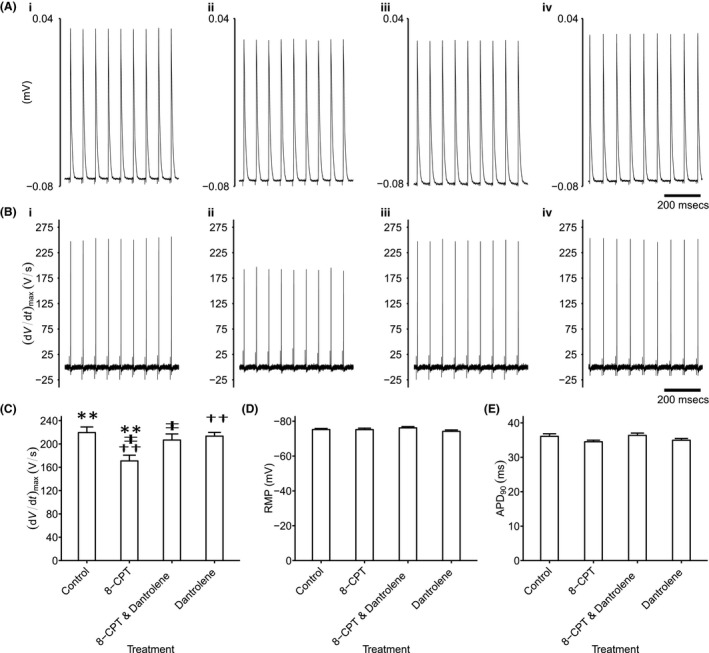
Analysis of atrial action potential waveforms. Intracellular recording of atrial action potential waveforms during regular 6 Hz pacing (A) and their first derivatives reflecting (d*V*/d*t*) (B) in (i) untreated cardiomyocytes, (ii) with 8‐CPT challenge followed by (iii) further addition of dantrolene, as well as (iv) dantrolene alone. These recordings yielded values of (d*V*/d*t*)_max_ (C) that could be compared with remaining features of the resting potential (RMP) (D) and APD_90_ (E). Single, double and triple symbols denote significantly different pairs of values, to a significance value of *P* < .05, .01 and .001 respectively

**Figure 8:**
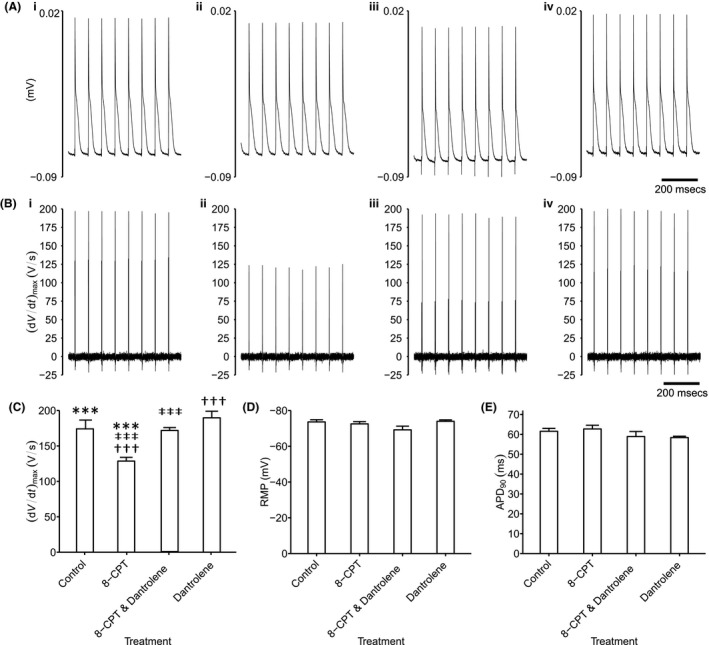
Analysis of ventricular action potential waveforms. Intracellular recording of ventricular action potential waveforms during regular 6 Hz pacing (A) and their first derivatives reflecting (d*V*/d*t*) (B) in (i) untreated cardiomyocytes, (ii) with 8‐CPT challenge followed by (iii) further addition of dantrolene, as well as (iv) dantrolene alone. These recordings yielded values of (d*V*/d*t*)_max_ (C) that could be compared with remaining features of the resting potential (RMP) (D) and APD_90_ (E). Single, double and triple symbols denote significantly different pairs of values, to a significance value of *P *<* *.05, .01 and .001 respectively

Reductions of Na^+^ current of the kind described above have been associated with corresponding reductions in maximum action potential upstroke rate, (d*V*/d*t*)_max_ in turn resulting in the pro‐arrhythmic slowing of AP conduction^32^ reported in previous explorations of effects of Epac modulation.^19^ Figures 7 and 8 illustrate typical traces of atrial and ventricular action potential waveforms in response to regular 6 Hz pacing (A) and their first derivatives whose peaks reflect (d*V*/d*t*)_max_ (B). In comparison with (i) untreated atrial (Figure 7) and ventricular cardiomyocytes (Figure 8), (ii) 8‐CPT challenge reduced (d*V*/d*t*)_max_. This was reversed by further addition of (iii) dantrolene, which when applied by itself (iv) similarly gave (d*V*/d*t*)_max_ resembling those of untreated hearts.

Significant differences in both atrial and ventricular (d*V*/d*t*)_max_ values were thus detected amongst findings obtained in the absence (atria: 219.56 ± 9.64 V/s [n = 23]; ventricles: 174.11 ± 6.21 V/s [n = 17]), and following challenge with 8‐CPT before (atria: 170.97 ± 9.78 V/s [n = 20]; ventricles: 128.64 ± 5.26 V/s [n = 14]), and following further addition of dantrolene (atria: 206.74 ± 10.47 V/s [n = 22]; ventricles: 170.90 ± 4.16 V/s [n = 20]) and of dantrolene applied alone (atria: 213.48 ± 6.39 V/s [n = 29]; ventricles: 189.70 ± 9.28 V/s [n = 20]) (atria: *F* = 5.41; *P *=* *.0018; ventricles: *F* = 12.98; *P *=* *8.8 × 10^−7^). There were significant pairwise (d*V*/d*t*)_max_ differences in cardiomyocytes studied before, and following 8‐CPT challenge (atria: *P *=* *.002; ventricles: *P *=* *.0002) but not following further addition of dantrolene (atria: *P *=* *.75; ventricles: *P *=* *.99) or addition of dantrolene alone (atria: *P *=* *.96; ventricles: *P *=* *.36). Thus, further addition of dantrolene restored Na^+^ current previously reduced by 8‐CPT (atria: *P *=* *.042; ventricles: *P *=* *.0004; Figures 7C and 8C). The similar findings in both atrial and ventricular preparations thus paralleled corresponding observations in loose patch clamp Na^+^ currents. In contrast, resting membrane potentials showed no significant differences between groups (atria: *F* = 1.24, *P *=* *.30; ventricles: *F* = 1.18, *P *=* *.32), falling close to −75 mV in both atrial and ventricular cardiomyocytes (Figures 7D and 8D). Similarly, action potential durations at 90% recovery showed no significant differences between experimental groups (atria: *F* = 2.10, *P *=* *.11; ventricles: *F* = 1.24; *P *=* *.296), falling close to ~35 and ~60 ms in atrial and ventricular cardiomyocytes respectively (Figures 7E and 8E).

## 3 DISCUSSION

The experiments here investigated for acute effects of Epac activation, known to acutely perturb cellular Ca^2+^ homeostasis,^18^ upon Na^+^ currents in wild‐type murine cardiomyocytes. The loose patch clamp method detected current flowing into an electrode apposed to the extracellular surface of a fixed area of intact cell membrane, yielding membrane currents normalised to the area of voltage‐clamped cell surface (pA/μm^2^). In contrast, conventional patch clamp methods typically normalise measured currents to background membrane capacitance (pA/pF). Atrial and ventricular cells showed differing Na^+^ current magnitudes (e.g. ~−42.6 ± 2.9 vs −27.5 ± 1.8 pA/pF;^39^ ~−89.59 ± 41.05 vs −50.20 ± 3.34 pA/pF respectively^40^), but total membrane capacitances differing in the opposite direction (~43 vs ~90 pF).^39^ Their total capacitances also differ in their relative contributions of surface to tubular membrane^41^, ^42^ that in turn express differing channel densities.^43^, ^44^ Nevertheless, the loose‐patch clamp permitted cardiomyocyte recordings to be made in intact superfused atrial and ventricular preparations, avoiding disruptions of intracellular Ca^2+^ homeostasis necessitated by the isolation and intracellular Ca^2+^ chelation otherwise required in conventional whole‐cell patch‐clamp recordings.^30^, ^31^ Loose‐patch clamp recordings also allowed employment of in vivo rather than reduced extracellular [Na^+^] levels sparing Na^+^‐Ca^2+^ exchange. Previous reports had identified early inward currents obtained with this technique with Na^+^ currents mediating action potential (AP) conduction and upstroke.^21^

The experiments followed directly from findings that acute Epac (exchange protein directly activated by cAMP) activation: (i) produced phosphokinase A (PKA)‐independent activation of RyR2‐mediated Ca^2+^ release,^13^, ^14^, ^15^ increasing Ca^2+^ spark frequencies in adult rat cardiomyocytes,^16^ Ca^2+^‐dependent Ca^2+^ release amplitudes after isoproterenol treatment,^17^ and amplitudes and frequencies of spontaneous Ca^2+^ release in mouse ventricular cardiomyocytes.^18^ It correspondingly, (ii) increased incidences of both triggered activity and ventricular tachycardia (VT).^18^ However, it also (iii) associated such pro‐arrhythmic effects with decreased conduction velocities.^19^ Furthermore, (iv) these electrophysiological characteristics were rescued by the RyR2‐Ca^2+^ release channel blocker dantrolene, despite (v) all these changes being associated with unaltered recovery characteristics as reflected in the cardiac action potential (AP) durations and ventricular effective refractory periods.^18^, ^19^

Such findings suggest that acutely altered Ca^2+^ homeostasis might result in arrhythmic substrate arising from delayed conduction, as previously suggested for the pro‐arrhythmic Brugada syndrome^1^, ^2^, ^3^, ^4^, ^5^ in contrast to the recovery abnormalities associated with the LQT syndromes.^6^, ^45^ The pro‐arrhythmic effects of acute abnormalities in cardiomyocyte Ca^2+^ homeostasis provoked by adrenergic stimulation, caffeine‐mediated RyR2 stimulation^10^ or modified extracellular Ca^2+^ entry,^11^, ^12^ have hitherto been primarily associated with arrhythmic triggering by delayed afterdepolarisation effects arising from the consequently altered Na^+^–Ca^2+^ exchanger activity.

Early Na^+^ current is a primary determinant of cardiac AP conduction velocity. The present experiments are the first time Na^+^ current measurements have been made during Epac activation using an experimental design that permits comparisons with those previous studies.^20^, ^21^, ^26^ Thus, in addition to employing similar methods of Na^+^ current measurement: (i) They also (cf.^18^) investigated effects of Epac activation using the agonist 8‐CPT^13^ at ~1 μmol/L, a concentration range known to provide a 300‐fold preferential selectivity for Epac over PKA pathways;^13^, ^33^, ^34^ 8‐CPT only inhibits phosphodiesterase isoforms at considerably higher concentrations.^35^ (ii) The reversibility of the pharmacological effects of 8‐CPT were tested for by the RyR2‐inhibiting agent dantrolene (cf.^19^) thought to stabilize RyR2 closed states by enhancing interactions between its N‐terminal and the central domains particularly under conditions of increased open channel probability.^36^, ^37^, ^46^ Dantrolene thus inhibits RyR2‐mediated diastolic Ca^2+^ release and decreases frequencies and durations of aberrant Ca^2+^ sparks in cardiomyocyte models for CPVT^36^ and cardiac failure.^37^, ^38^ (iii) Effects of dantrolene were further controlled for in experiments involving direct exposure to dantrolene, both by itself and in combination with 8‐CPT (cf.^19^), and (iv) The experiments similarly measured the time courses and steady‐state voltage‐dependences of Na^+^ current activation and inactivation (cf.^21^), in both atrial and ventricular preparations from WT murine hearts.

The variation of the test step to voltage *V*_1_ allowed measurement of the peak amplitudes of Na^+^ current and yielded current‐voltage activation relationships. These peaks were followed by decays to a steady‐state inactivation level whose dependence upon the voltage *V*_1_ was assessed by further voltage steps to a standard depolarised voltage *V*_2_. 8‐CPT markedly reduced the Na^+^ currents in both procedures. The inhibitory effect of 8‐CPT was reversed by further addition of dantrolene. In contrast, transfer of cardiomyocytes from pretreatment conditions to solutions containing dantrolene alone or dantrolene combined with 8‐CPT left the Na^+^ currents intact. Furthermore, detailed parameters describing the voltage dependence of current inactivation were unchanged through these pharmacological conditions, as were the time courses of recovery from such inactivation.

These findings thus demonstrate that acutely altered Ca^2+^ homeostasis following Epac‐mediated RyR2‐activation reversibly reduces Na^+^ channel function. Previous reversible manipulations of loose patch pipette [Na^+^] had identified early inward currents following step depolarisations with cardiomyocyte Na^+^ currents driving the maximum upstroke rate, (d*V*/d*t*)_max_, and in turn conduction of the cardiac action potential^21^. The present experiments accordingly went on to investigate such corresponding changes in action potential activation reflected in (d*V*/d*t*)_max_ measurements from intracellular sharp electrode recordings of membrane potential in intact atrial and ventricular preparations under comparable pharmacological conditions. The observed (d*V*/d*t*)_max_ were reduced with 8‐CPT challenge; this effect was absent with dantrolene whether applied alone or in combination with 8‐CPT. This was in direct agreement with the observed changes in Na^+^ currents under corresponding pharmacological conditions. Together these findings implicate such Na^+^ current alterations with the pro‐arrhythmic reductions in AP conduction velocity observed in previous explorations of the effects of Epac modulation in murine hearts.^19^

The present findings thus implicate conduction velocity secondary to compromised Na^+^ current as a source for arrhythmic substrate under conditions of *acutely* perturbed cytosolic Ca^2+^ homeostasis, reconstructing the altered conduction previously reported in Na_v_1.5 haplo‐insufficient, *Scn5a* ^+/−^ murine models for Brugada Syndrome.^1^, ^2^, ^3^, ^4^, ^5^ They also complement previous findings in murine hearts chronically modelling catecholaminergic polymorphic ventricular tachycardia (CPVT). Cardiomyocytes in the latter systems similarly showed diastolic episodes, or propagating waves, of RyR2‐mediated Ca^2+^ release, as well as afterdepolarisation and triggering phenomena.^8^, ^9^ *RyR2*‐P2328S hearts additionally showed parallel reductions in atrial^21^ and ventricular action potential conduction velocities, the latter particularly following catcholaminergic challenge.^20^ These changes accompanied chronically downregulated Na_v_1.5 expression.^21^, ^27^ Furthermore, WT rat cardiomyocytes increased their expression of functionally active surface membrane Na_v_1.5, Na_v_1.5 mRNA and total Na_v_1.5 protein following verapamil challenge and decreased their surface membrane Na_v_1.5 expression following calcimycin challenge.^47^, ^48^ In parallel with the present findings, *RyR2*‐P2328S cardiomyocytes also demonstrated acutely reduced Na_v_1.5 function.^20^, ^21^, ^26^ This was partially rescued by pharmacological interventions reducing RyR2‐mediated Ca^2+^ release.^22^, ^23^, ^24^, ^25^ These findings together suggest direct effects of altered cytosolic [Ca^2+^] upon Nav1.5 function.^49^, ^50^, ^51^ Certainly, patch‐clamped WT myocytes show respective reductions, or increases in Na^+^ current and (d*V*/d*t*)_max_, with increases in, or sequestration of, the pipette [Ca^2+^].^52^ Structural evidence suggests direct and/or indirect Ca^2+^ binding sites on Nav1.5 whose occupancy might modify Nav1.5 channel function. Close to the Nav1.5 carboxy‐terminal, direct Ca^2+^ binding occurring at an EF hand motif may increase Na^+^ channel activity.^53^ In contrast, an additional indirect ‘IQ’ domain binding site permits Ca^2+^/calmodulin (CaM) binding. Finally, multiple phosphorylatable sites in the DI‐II linker region, including serines 516 and 571, and threonine 594, are targeted by calmodulin kinase II (CaMKII)^54^, ^55^, ^56^. The latter two binding mechanisms require prior Ca^2+^ binding to EF hand motifs in Ca^2+^/CaM or CaMKII and compromise Na^+^ channel activity.^50^, ^51^

## 4 MATERIALS AND METHODS

### 4.1 Solutions

Krebs–Henseleit (KH) solution was prepared (mmol/L: NaCl, 119; NaHCO_3_, 25; KCl, 4.0; KH_2_PO_4,_ 1.2; MgCl_2,_ 1.0; CaCl_2,_ 1.8; glucose, 10; and Na‐pyruvate, 2.0; pH adjusted to 7.4 and bubbled with 95% O_2_/5% CO_2_ (British Oxygen Company, Manchester, UK)) for all tissue preparations. Chemical agents were purchased from Sigma‐Aldrich (Poole, UK) unless otherwise stated. A modified KH solution containing 10 mmol/L 2,3‐butanedione monoxime (BDM) and 10 μmol/L blebbistatin (Cayman Chemical Company, Ann Arbor, MI, USA) was also prepared for Langendorff perfusion of isolated hearts to electromechanically uncouple the heart prior to isolation of right ventricular preparations. KH solution containing 8‐(4‐chlorophenylthio)adenosine 3′,5′‐cyclic monophosphate sodium salt (8‐CPT, 1 μmol/L) and/or dantrolene (10 μmol/L) (LKT Laboratories Inc, St Paul, MN, USA) was prepared and filtered to remove particles greater than 10 μm in diameter with standard filtration paper (Millipore, Bedford, MA, USA).

### 4.2 Tissue preparation

This research has been regulated under the Animals (Scientific Procedures) Act 1986 Amendment Regulations 2012 following ethical review by the University of Cambridge Animal Welfare and Ethical Review Body (AWERB). It also conformed to the Guide for the Care and Use of Laboratory Animals, U.S. National Institutes of Health (NIH Publication No. 85‐23, revised 1996). C57BL6 WT mice aged 3‐6 months were used in all experiments. Mice were housed at room temperature in a licensed facility, allowed free access to sterile rodent chow and water, and subjected to 12 hours light/dark cycles. Mice were killed by cervical dislocation (Schedule 1 of the UK Animals (Scientific Procedures) Act 1986), following administration of 200 IU intraperitoneal heparin (ACROS Organics. ThermoFisher, Loughborough, Leicestershire, UK) 10 minutes prior to death. The heart was immediately excised and transferred into ice‐cold KH solution. The aorta was cannulated with a trimmed and filed 21G hypodermic needle, and secured onto the cannula with an aneurysm clip and 5‐0 braided silk suture. The heart was then perfused retrogradely in a Langendorff system under constant flow (2 mL/min) by a Watson‐Marlow (Falmouth, UK) peristaltic pump with 75 mL KH‐BDM/blebbistatin solution to electromechanically uncouple the heart. Following cessation of contractions, the heart was immediately transferred into ice‐cold KH‐BDM/blebbistatin solution. For the loose patch clamp experiments, the atria and right ventricle were dissected from the rest of the heart, mounted onto Sylgard (Dow Chemical Company, Staines, UK) then placed in the bath containing filtered KH buffer solution maintained at 27°C. Experiments performing intracellular electrophysiological measurements transferred the hearts to a Langendorff perfusion system for the electrophysiological studies described below.

### 4.3 Loose patch clamp recording

Pipettes were pulled from borosilicate glass capillaries (GC150‐10 specification Harvard Apparatus, Cambourne, Cambridge, UK) using a micropipette puller (Flaming/Brown micropipette puller; model P‐97, Sutter Instrument Co. Novato, CA, USA). Pipettes were then mounted under a microscope at 250× magnification with a calibrated eyepiece graticule, and fractured with a diamond knife under visual control. Transverse force applied to the distal tip of the pipette resulted in fracture perpendicular to the axis of the micropipette. Selected pipettes were fire‐polished using an electrically heated nichrome filament under visual guidance at 400× magnification. The pipette tips were then bent to ~45° from the shaft of the pipette to allow the membrane to be approached vertically when mounted on the recording amplifier headstage. Maximum internal tip diameters were measured at 1000× magnification. All pipettes were of 28‐32 μm in diameter following polishing.

The distal half of the micropipettes were filled with KH buffer and mounted onto a pipette holder connected to a headstage. Connection of an air filled line to the pipette holder allowed for application of suction through a syringe to form membrane seals. Ag/AgCl electrodes maintained electrical connections to the organ bath and pipette. Loose patch‐clamp experiments were performed as previously described.^21^, ^24^, ^27^ The pipette was lowered onto the membrane surface and gentle suction applied to allow seal formation around the patch of membrane. Voltage‐clamp steps were delivered under computer control relative to the resting membrane potential. The loose patch clamp method applies voltage steps to the extracellular surface of membrane within the seal. Positive and negative voltage steps applied through the pipette respectively hyperpolarise and depolarise the membrane potential relative to the initial cell resting membrane potential (RMP). In the text membrane potentials are therefore stated in terms of their displacement from the RMP in the same convention adopted by earlier studies using this technique.^29^, ^57^ The loose patch clamp configuration results in larger leakage currents than the conventional patch clamp due to the comparatively low seal resistance. A custom‐built amplifier was used to compensate for the majority of the leakage current, series resistance errors and the displacement current through the pipette capacitance.^28^ Residual leakage and capacitative currents were corrected for using reference records from subsequent P/4 control protocols applying steps whose amplitudes were scaled down by a factor of four and of the opposite sign relative to the test steps, as fully described previously.^29^, ^57^

Experiments were first performed under pretreatment conditions using preparations superfused with KH solution. Measurements were then made with KH solution containing 8‐CPT (1 μmol/L), then with KH solution containing both 8‐CPT (1 μmol/L) and dantrolene (10 μmol/L). To control for effects of dantrolene, further experiments superfused preparations with KH solution containing dantrolene (10 μmol/L) both alone and in combination with 8‐CPT (1 μmol/L). Patch‐clamp studies took place under standardised electrophysiological conditions following similar intervals after each solution change. Each patch was subject only to a single application of the pulse protocols. These made differences between results attributable to prolonged changes in the patch such as bleb formation unlikely.^58^

### 4.4 Whole heart intracellular microelectrode recordings

Microelectrode studies were performed using a modified horizontal Langendorff perfusion system incorporating a light microscope (objective ×5, eyepiece ×5, W. Watson and Sons Limited, London, UK), and warmed tissue chamber housed within a Faraday cage. Stimulating and recording electrodes were positioned on the heart using micromanipulators (Prior Scientific Instruments, Cambridge, UK). Sharp microelectrodes drawn from borosilicate glass pipettes (OD 1.2 mm, ID 0.69 mm, Harvard Apparatus, Cambridge, UK) using a homebuilt microelectrode puller, filled with 3 M KCl (tip resistance 15‐25 MΩ), and mounted onto a right‐angled microelectrode holder containing a Ag/AgCl half‐cell were connected to a high‐input impedance direct‐current microelectrode amplifier system (University of Cambridge, Cambridge, UK). Following band‐pass filtering (between 0 and 2 kHz), signals were subject to analogue‐to‐digital conversion for recording at a sampling frequency of 10 kHz (1401; Spike software: Cambridge Electronic Design, Cambridge, UK). Impalements were consistently made in the proximal left ventricle to minimise confounds from regional variations in AP characteristics. Intracellular voltages were measured relative to that of a bath Ag/AgCl reference electrode. Impalements were used for detailed recordings where they resulted in abrupt appearance of a resting membrane potential (RMP) between −65 and −90 mV and APs of normal amplitude >75 mV. Hearts were paced by a bipolar platinum‐coated stimulating electrode (NuMed, New York, USA) applied to the right ventricle lateral surface using a DS2A‐Mk.II stimulator (Digitimer, Welwyn Garden City, Herts., UK) applying a stimulus voltage twice diastolic excitation threshold plus 0.5 mV, at a regular 6 Hz pacing frequency. Maximum rates of action potential depolarisation (d*V*/d*t*)_max_ were obtained from the first time differential of the intracellular AP waveform. These were measured along with cardiomyocyte resting potentials, RMP, with AP amplitude measured from the RMP to the peak AP voltage. Action potential duration was measured as the time from the AP peak to 90% repolarisation to baseline.

### 4.5 Analysis of results

The currents obtained from the loose patch procedure are dimensioned in units of current (nA). Subsequent analysis converted the units of current (nA) to current densities (pA/μm^2^) using the formula:

$$\text{Current density}=\frac{1000\times \text{current}}{\left({\pi \times \text{pipette}\;\text{radius}}^{2}\right)}$$

Current‐voltage curves describing Na^+^ current activation and inactivation and current‐time relationships plotting the recovery timecourse of Na^+^ current from inactivation were obtained from the corresponding measurements of peak Na^+^ currents (means ± standard error of the mean [SEM]) in the relevant protocol (see Figure 1Aa). Peak Na^+^ currents, *I*, reflecting inactivation properties were empirically related to the inactivating voltage *V *= *V*_1_ by a Boltzmann function: *I *= *I*_max_/{1 + exp(*V*−V*/*k*)}. Peak currents obtained during determinations of the timecourse, *T*, of recovery from inactivation were described by the exponential function *I *= *I*_max_(1−exp(−*T*/τ)). The curve‐fitting procedures employed the open source fitting algorithms QtiPlot (Version 0.9.9‐rc9).

### 4.6 Statistical analysis of results

Owing to the limited recording time permitted following establishment of each loose patch,^58^ each pharmacological condition was tested using different patches giving unpaired comparisons of results obtained (i) before, and following (ii) 8‐CPT treatment, (iii) 8‐CPT followed by addition of dantrolene, (iv) 8‐CPT combined with dantrolene, and (v) dantrolene alone initially using one‐way analysis of variance (ANOVA) to an α < .05 significance level. Subsequent posthoc comparisons suggested by a presence of significant differences employed a Bonferroni method in view of its application to (i) a subset of four rather than all possible pairwise comparisons with (ii) the number of contrasts to be estimated close to the number of factors (*NIST/SEMATECH e‐Handbook of Statistical Methods*, http://www.itl.nist.gov/div898/handbook/).

## DISCLOSURE

None declared.
